# Filament assembly of the *C. elegans* lamin in the absence of helix 1A

**DOI:** 10.1080/19491034.2022.2032917

**Published:** 2022-02-07

**Authors:** Rebecca de Leeuw, Rafael Kronenberg-Tenga, Matthias Eibauer, Ohad Medalia

**Affiliations:** Department of Biochemistry, University of Zurich, Zurich, Switzerland

**Keywords:** C. elegans, intermediate filaments, lamins, cryo-electron tomography

## Abstract

Lamins are the major constituent of the nuclear lamina, a protein meshwork underlying the inner nuclear membrane. Nuclear lamins are type V intermediate filaments that assemble into ~3.5 nm thick filaments. To date, only the conditions for the *in vitro* assembly of *Caenorhabditis elegans* lamin (*Ce*-lamin) are known. Here, we investigated the assembly of *Ce*-lamin filaments by cryo-electron microscopy and tomography. We show that *Ce*-lamin is composed of ~3.5 nm protofilaments that further interact *in vitro* and are often seen as 6–8 nm thick filaments. We show that the assembly of lamin filaments is undisturbed by the removal of flexible domains, *that is,* the intrinsically unstructured head and tail domains. In contrast, much of the coiled-coil domains are scaffold elements that are essential for filament assembly. Moreover, our results suggest that *Ce*-lamin helix 1A has a minor scaffolding role but is important to the lateral assembly regulation of lamin protofilaments.

## Introduction

**Figure 1.**
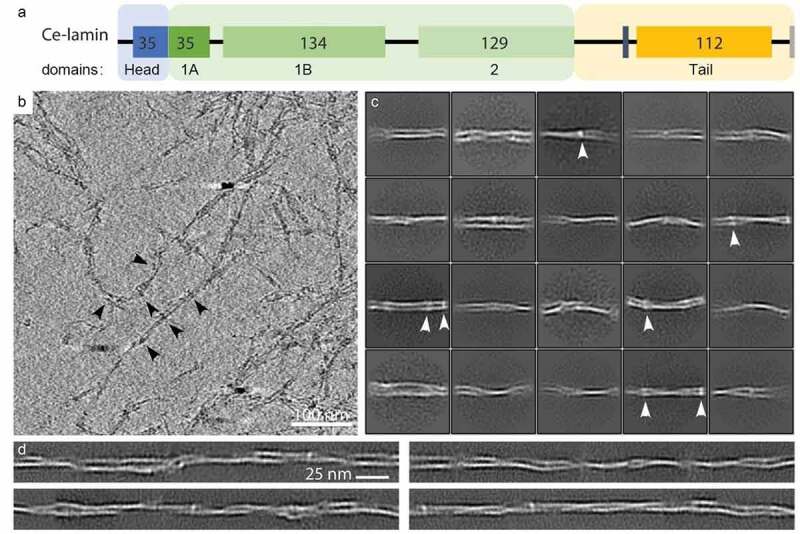
Cryo-ET analysis of the *C. elegans* lamin filaments. (a). A schematic view of the *Ce-lamin* protein , indicating the different domains of the protein. The intrinsically disordered head domain (blue), is composed of 48 amino acids. The first 14 aa are shown as a black line, followed by 35 conserved aa which are found in other organisms. The coiled-coil rod domain (large green box) is composed of the canonical helix segments and their linkers. Helix 1A (35 aa), helix 1B (134 aa) and helix 2 (129 aa) are shown. The tail domain (yellow) is composed of an Ig-like domain (112 aa) flanked by unfolded protein stretches (black lines). The positions of the nuclear localization sequence (vertical gray stripe) and the CaaX motif (vertical light gray stripe) are indicated. (b). The filaments are detected in an x-y slice through a cryo-electron tomogram, 3 nm in thickness. Arrowheads point to some of the globular domains seen along the filaments. Scale bar 100 nm. (c). Structural class averages of *in silico* segmented filaments, extracted from cryo-tomograms with a box size of 70^2^ nm^2^. The filaments appears two protofilaments (horizontaly oriented) that are often bent. This appearance indicates the flexibility of the filaments. Additional densities, presumably Iglike domains, are often seen (arrowheads). (d). *In silico* reconstituted filaments, from the structural class averages shown in c., indicate the flexibility and interactions between the protofilaments.

The nuclear lamina is a proteinaceous meshwork layer that provides structural and mechanical support at the nuclear phase of the inner nuclear membrane (INM). Its main constituents are assembled nuclear lamins that form a dense meshwork bridging between the INM and chromatin [1,2]. Lamins play a major role in conferring special mechanical properties to the nucleus, due to the unique mechanical characteristics of the assembly [3–6]. Based on their amino acid sequence, lamins have been classified as the ancestors of the intermediate filaments (IFs) family of proteins [7]. They are composed of a central α-helical coiled-coil domain, flanked by a non-α-helix N-terminal head and globular tail domains [8,9]. The rod domain is comprised of four coiled-coil α-helical segments, helices 1A, 1B, 2A, and 2B, which are comprised of the typical periodic heptad repeats. The lamin tail domain hosts the nuclear localization signal and a highly conserved immunoglobulin (Ig) like fold, flanked by additional unstructured amino acid stretches (Figure 1a).

The *Caenorhabditis elegans* lamin (*Ce*-lamin) is a single lamin isoform in which the head domain is composed of 48 amino acids, 14 amino acids more than its mammalian homologues. Early electron microscopy studies revealed the basic steps involved in lamin assembly. The elementary building block of lamins is a polar elongated dimer, consisting of a ~50 nm long rod-like structure, flanked by two non-α-helical head and tail domains, packed in close proximity [10]. The two coiled-coil α-helical domains interact in a parallel fashion and further assemble into a polar head-to-tail polymer of dimers. The head-to-tail polymers of lamin dimers can further associate laterally [11], presumably in an antiparallel fashion, to form the mature lamin filament [12]. High-resolution structural analysis of lamin A fragments suggested that the interactions driving head-to-tail polymer assembly involve the interaction between helix 1A of a dimer with the helix 2B of the subsequent dimer [13]. In contrast, structural analysis of the N-terminal half of lamin A, suggested that tetramers are the polymerizing building blocks [14].

Four main lamin isoforms are found in mammals, encoded by the *LMNA, LMNB1*, and *LMNB2* genes that encode the lamin A/C, lamin B1 and lamin B2 proteins, respectively [15]. The B-type lamins are expressed in almost all mammalian cell types, as well as in *C. elegans*. Thus, the *Ce*-lamin is a B-type lamin encoded by the *lmn-1* gene [16]. Like other B-type lamins, the *Ce-*lamin is constitutively farnesylated. However, the helix 2B coil segment is found to be shortened by two heptad repeats, as compared to vertebrate lamins. Interestingly, the helix 1A domain lacks a phosphorylation site that is found in lamin A. Moreover, *Ce*-lamin has one of the shortest tail domains of any lamin protein, which is 179 amino acids in length.

*Ce*-lamin is the only known lamin that can be assembled *in vitro* into stable filaments [17,18]. Therefore, it was previously subjected to cryo-electron tomography (cryo-ET) analysis, which indicated that the lamin filaments are composed of protofilaments that interact with each other to form stable filaments [19,20]. The protofilaments, ~3.5 nm in diameter, resemble the lamin assembly in mouse embryonic fibroblasts [21,22]. These filaments were also produced and analyzed by ectopic expression in *Xenopus* oocytes, which indicated that ~3.5 nm thick protofilaments are the basic building blocks of *Ce*-lamin [23]. Furthermore, ~3.5 nm thick filaments were detected in vitrified *C. elegans* embryos by cryo-ET [24].

Here, we expressed and assembled *Ce*-lamin into filaments *in vitro*. Using cryo-electron microscopy (cryo-EM) and tomography in conjunction with averaging approaches, we gain insights into its organization. We followed the filaments by *in silico* reconstitutions from class averages. Next, we expressed and assembled *Ce*-lamin in the absence of its tail, head, and helix 1A domains. These experiments indicate that *Ce*-lamin assembles in the absence of these domains and suggest that helix 1A is responsible for restricting the lateral assembly of fully assembled *Ce*-lamin filaments.

## Material and methods

### Protein expression and purification

Wild-type *lmn-1* gene (amino acid residues 1–556), tailless *Ce*-lamin (residues 1–385), headless *Ce*-lamin that is also deficient in the last 15 amino acids beyond the Ig-fold (residues 48–551), and *Ce*-lamin lacking coil 1A, coil 1B, or coil 2, respectively, were cloned into the pET24d vector, with an N-terminal histidine tag and TEV (Tobacco Etch Virus) protease site [17] and expressed in *E. coli* LOBSTR competent bacteria [25] (wild-type) or Rosetta(De3)pLysS competent bacteria. All mutations were carried out using mutagenesis PCR, the QuikChange Lightning Site-Directed Mutagenesis Kit (Agilent Technologies), see Supplementary Information Table 1. The proteins were expressed and purified as described in Foeger *et* *al*., [17]. The bacterial cultures were incubated at 37°C and expression was induced by 0.3–1 mM IPTG (Isopropyl β- d-1-thiogalactopyranoside) for 3–4 hours at 37°C. Bacteria were harvested by centrifugation at 4000 g for 20 minutes. The *Ce*-lamin proteins were purified as previously described [26]. Bacterial pellets were rapidly thawed and resuspended in RS buffer (20 mM Tris-HCl, pH 7.5, 200 mM NaCl) supplemented with 200 µg/ml lysozyme and cOmplete protease inhibitor cocktail (Roche). 1 U/ml DNaseI (AppliChem) and 3 mM final concentration MgCl_2_ were added and the suspension was incubated 15 minutes on ice. The resuspended bacteria were broken via sonication and the crude extract was centrifuged at 4000 g for 10 minutes. The pellet was washed in RS buffer supplemented with 1% (w/v) Triton X-100. The pellet was washed twice in RS buffer and resuspended in urea buffer (8 M urea, 50 mM Tris-HCl, pH 7.5, 200 mM NaCl, 1 mM DTT) and centrifuged at 10.000 rpm (SS-34 rotor) for 10 minutes. The supernatant was diluted in 20 mM Tris-HCl, pH 8.0, 2 mM MgCl_2_, 1 mM PMSF, 20 mM NaCl, supplemented with 250 U/ml Benzonase (Merck Millipore) and incubated for 30 minutes. Next, centrifugation was repeated and the pellet was resuspended in 20 ml binding buffer (8 M urea, 50 mM Tris-HCl, pH 8.0, 500 mM NaCl, 20 mM imidazole), which was incubated with Ni-NTA beads (Qiagen). The beads were loaded onto a bench-top column and washed. Elution buffer (8 M urea, 20 mM Tris-HCl, pH 7.5, 100 mM NaCl, 200 mM imidazole) was added and left to settle into the beads for 10 minutes. The concentration of the protein was monitored by UV spectrophotometry. The fractions containing *Ce*-lamin were pooled and dialyzed against 500 × 8 M urea, 20 mM Tris-HCl, pH 8.0, 20 mM NaCl. The samples were further purified using an anion exchanger, using 500 µl DEAE beads. The beads were washed with 20 mM KCl, 20 mM Tris‐HCl, pH 8.0, 8 M urea, and eluted using 20 nm KCl step gradient from 20 mM to 400 mM KCl, with 20 mM steps and 500 µl buffer. Protein content was assessed via Bradford staining. Fractions containing *Ce*‐lamin were pooled and dialyzed overnight against 1000 × 8 M urea, 20 mM Tris‐HCl, pH 8.0.     

### Assembly of Ce-lamin filaments

Bacterially expressed and purified *C. elegans* lamin (0.05–0.1 mg/ml) in urea buffer was dialyzed at 4°C against 1000 ml buffer containing 2 mM Tris-HCl, pH 9.0, 1 mM DTT for 4 h, followed by 16 h dialysis against a buffer containing 15 mM Tris-HCl, pH 7.4, 1 mM DTT.

### Negative stain transmission electron microscopy

Carbon coated grids were glow-discharged prior to sample application. 3 µl samples were applied to glow-discharged carbon coated copper grids and negatively stained with 1% (w/v) uranyl acetate (Fluka). The grids were inspected by a FEI Tecnai G2 Spirit 120 kV transmission electron microscope with a digital CCD camera.

### Cryo-electron microscopy and tomography

A 3 μl drop of assembled *Ce*-lamins was applied onto a glow-discharged 200-mesh carboncoated copper grid (Quantifoil, Jenna, Germany), which was coated with an additional layer of ~2 nm carbon, and vitrified immediately. For cryo-ET, fiducial 10 nm BSA gold tracer markers (Aurion) were added prior to vitrification. Data was collected using a Titan Krios G^2^ (Thermo Scientific) electron microscope equipped with a Gatan Quantum Energy Filter with a K2 or K3 direct electron detector. Images were acquired in counting mode at a magnification of 81.000 x, resulting in a pixel size of 1.7^2^ Å^2^. Images of *Ce*-lamin Δcoil 1A filaments were collected with a pixel size of 81.000 x, resulting in a pixel size of 1.06^2^ Å^2^, using a K3 detector.

2997 images were collected with a defocus range between 1.8 and 2.8 µm underfocus. Tomograms were acquired using the SerialEM software package [27] in a bidirectional fashion, starting at −30°. Tilt series were collected between −60° and 60°, with 3° interval at a defocus of −4 µm. Each tilt movie was exposed for 1.4 s in 0.2 s frames; with 1.95 e^−^/Å^2^ per tilt and a total dose of 112 e^−^/Å^2^. Tilt series were collected with a flux of 9.6 e^−^/pixel/s, at a magnification of 64.000 x and a corresponding pixel size of 2.2^2^ Å^2^.

### Image processing

Micrographs were CTF-estimated using Gctf [28] and processed in RELION 3.0.8 [29]. Further image analysis was performed using MATLAB. 94,811 filament stretches of 60 nm × 60 nm were acquired for *Ce*-lamin Δcoil 1A and were subjected to a 2D averaging procedure using a spherical mask, 40 nm in diameter.

Tomograms were reconstructed using the IMOD tomographic workflow [30] and CTF-corrected in MATLAB using the TOM toolbox [31]. Some tomograms were CTF-corrected using Ctfplotter [32]. The tomograms were automatically segmented using EMAN2 [33]. Segmentations were cleaned up using Chimera [34] and particle coordinates and particles were extracted in MATLAB.

Sixty-five tomograms were acquired for the *Ce*-lamin wildtype filaments and 100 tomograms for the ∆head-C∆15 mutant, which resulted in 54,955 and 361,448 lamin segments, respectively. The 2D projection images, 15 nm in thickness and with a size of 70 nm × 70 nm, were processed as previously described [11].

### *In silico* reconstituted filaments

Since the coordinates of each filament segment are known, using a reverse translation and rotation operator for each particle permits the reconstitution into a filament. We performed filament reconstitution for *Ce*-lamin wild-type and Δcoil 1A single-particles. We mapped back each particle to the micrograph by replacing the original particle within a filament with the respective class average it was sorted into. This was possible because each extracted particle was saved with its original coordinates. The filaments are reconstituted *in silico* [11,35]. To properly analyze reconstituted filaments, we also applied an unbending algorithm from ImageJ [36]. Firstly, a blurred mask is made to mask the overlap region between two segments along a filament, which in this case is 5 nm. Next, the mask is shifted to the center of the particle using the origin x and y coordinates. Then, the class average in which the particle was sorted is multiplied with the mask and re-centered. Next, the cropped segment is rotated around its rotational angle. The segments are placed in the same order as the straightened filament.

## Results and discussion

### *Ce*-lamin is assembled by multiple 3.5 nm thick protofilaments

Lamins are assembled in the nuclear lamina to form a dense meshwork with diverse functional aspects. Insight into the assembly and 3D structure of the lamin filaments is therefore of major importance. The *Ce*-lamin can be expressed in high quantities in bacteria, although it is localized to inclusion bodies [37]. Following a two-step dialysis, lamins were assembled into filaments. This methodology allows the study of *Ce*-lamin filament architecture and assembly *in vitro*. Although the bacterially expressed lamin carries no post-translational modifications, the *in vitro* assembled filaments resemble ectopically expressed *Ce*-lamin in *Xenopus* oocytes [23].

The *Ce*-lamin is a 556 amino acids (aa) protein, which is composed of a head domain, helix 1 (divided to 35 aa of helix 1A and 134 aa helix 1B), helix 2, and a tail domain (Figure 1a). Using cryo-ET, we set out to study the organization of the *Ce*-lamin filamentous structure. An x-y slice through a tomogram shows a dense filamentous network of lamin on the EM grid (Figure 1b). To gain insight into the structural organization of these filaments, we employed averaging approaches. These filaments appeared flexible, curved, and often interacted with neighboring filaments (Figure 1b). The globular densities that are decorating these filaments (Figure 1b, arrowheads) were presumably the Ig-like fold domains. These globular structures are seen along the filaments with variable distance and positioning. The amino acid linker between the end of the coiled-coil domains and the Ig-like fold domain, provides some flexibility in their position along the filaments. Next, we fragmented the filaments into 70 nm long segments, *in silico*, and calculated their 2D projections followed by a single-particle 2D classification approach [11,38]. Analysis of the most occupied 2D classes indicated different views of ~3.5 nm diameter protofilaments that interact to form ~6–8 nm thick filaments. These ~3.5 nm filaments often interact and cross each other and are sometimes seen as one structure (Figure 1c). The globular densities that are decorating these filaments, are seen in the 2D class averages as well (Figure 1c, arrowheads) although their appearance and position indicate a degree of flexibility along the filaments. Next, we mapped back the class averaged structures of *Ce-*lamin filaments to their original coordinates in the tomograms [11,39]. This approach allows us to resolve the reconstituted filaments at higher resolution and with higher contrast than in the original tomograms (Figure 1d). Two protofilaments compose the mature filaments. These protofilaments interact and cross each other frequently but seemingly without a strict pattern (Figure 1d). These sub-structures resembled the mammalian lamin filaments in shape and dimension [6,21]. The two filamentous structures often interacted with each other. It may suggest that the *Ce*-lamin filaments can form a higher ordered structure when exposed to different conditions.

### *Ce*-lamin filaments in the absence of the head and tail domains

**Figure 2.**
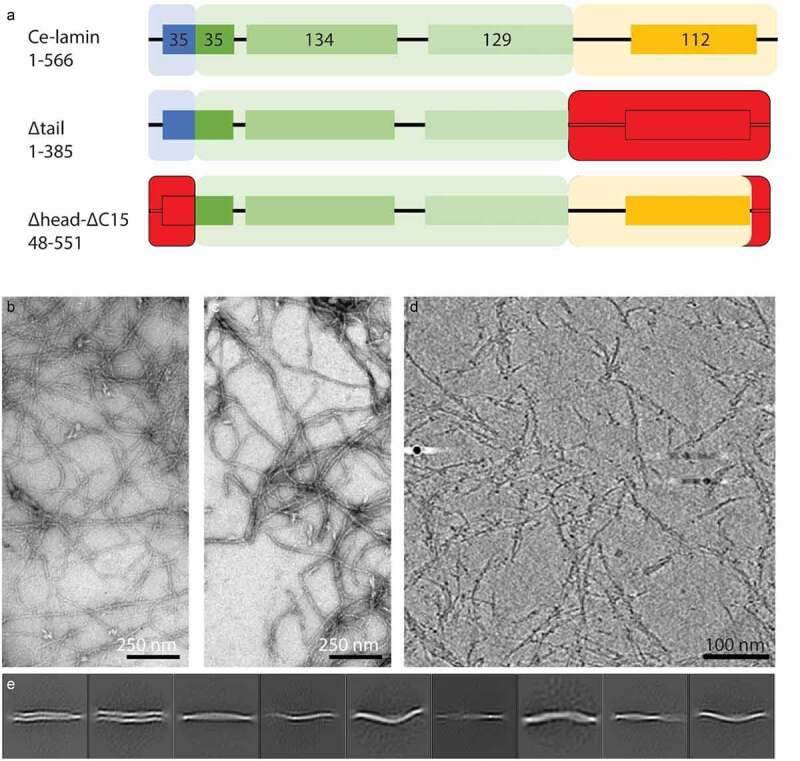
Lamin filament assembly in the absence of the head or tail domain. (a). Schematic illustration of the *Ce*-lamin protein, shows the deletions (red) of the tail domain (∆tail) or the head and the last 15aa (∆ead-∆C15). (b&c). Negatively stained electron microscopy images of the wild-type and ∆tail *Ce*-lamin filaments, respectively. (d). The ∆head-C∆15 lamin filaments were imaged by cryo-ET (a negatively stained electron microscopy image is shown in Figure S1C). An x-y slice through a cryo-tomogram shows the filaments and indicates their similarity to the wild-type filaments, as shown in Figure 1b. (e). Structural class averages of in silico segmented filaments extracted from tomograms, with a box size of 70^2^ nm^2^, resemble the structural class averages, as seen with the wild-type protein (Figure 1c).

The contribution of the head and tail domains to lamin assembly are controversial. While the *in vitro* assembly of lamin A/C was affected by deletion of the head domain, no effect was detected when both head and tail domains were removed [40]. In order to learn about the contribution of the head and tail domains to the *Ce*-lamin filament assembly, we expressed and assembled *Ce*-lamin truncation mutants that lack the head or the tail domain. *Ce*-lamin lacking the tail domain (∆tail) assembles into filaments and forms a dense meshwork resembling the wild-type *Ce*-lamin assembly (Figure 2b, C respectively). Next, we deleted the head domain and the presumably unstructured sequence beyond the Ig-like domain (Figure 2a, ∆head-∆C15). The assembled protein assembly was subjected to cryo-ET analysis. Figure 2d shows a typical slice through a tomogram, which shows the lamin filaments. The filaments resemble the wild-type assembly (Figure 1b, Figure S1C). We hypothesized that the deletion of presumably flexible, non-structured sequences would influence the flexibility of the lamin filaments. However, 2D class averages of these filaments indicated that the deletions do not have a major influence on the filament, and they do not have a substantial effect on the *Ce*-lamin assembly (Figure 2e).

Previous structural studies showed that the N- and C-terminal helical domains of lamin A can interact longitudinally [13]. Our results support these observations and suggest that the unstructured 48 aa long head domain of the *Ce*-lamin does not influence filament assembly. Similarly, the tail domain does not appear to be directly involved in filament assembly, but may have an important role in binding and anchoring lamins at the nuclear lamina.

### Lack of helix 1A promotes lateral association of *Ce*-lamin filaments

**Figure 3.**
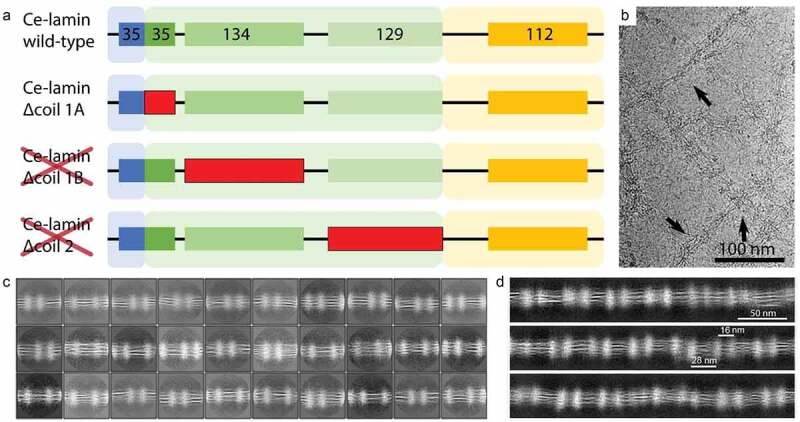
Helix 1A restricts lateral assembly of *C. elegans* lamin filaments, while helix 1B and 2 are indispensable. (a). Schematic illustration of the *Ce*-lamin and the deletion mutations (red) that were used. No filaments were detected by negative stained electron microscopy analysis of *Ce-*lamin Δcoil 1B and Δcoil 2. (b). A cryo-EM image of the *Ce*-lamin Δcoil 1A filaments (arrows). C. Structural class averages indicate several protofilaments and the repeating density clouds (oriented vertically). Horizontally oriented filaments are decorated by vertical cloud densities, presumably the Ig-like fold domains. (d). Reconstituted filaments from the *C*e-lamin Δcoil 1A cryo-EM analysis, showing an alternating pattern of 28 ± 2 nm and 16 ± 2 nm (these distances indicated in the figure). Scale bar 50 nm, indicated for C and D.

The coiled-coil domains of lamins serve as the scaffold of the lamin filaments. Here, we explored whether *Ce*-lamin can be assembled into filaments in the absence of helix 1 and 2. As expected, the *Ce-*lamin protein lacking helix 2, as well as helix 1B did not form any filaments (Figure 3a, Figure S1). However, when helix 1A was deleted, the protein assembled into thicker filamentous assemblies. We have acquired images of these assembled filaments using the cryo-EM single-particle analysis approach (Figure 3b). We used these images to obtain structural class averages (Figure 3c). The averaged classes indicate that the filaments are ~20 nm in thickness. 4–6 protofilaments were detected per filament (Figure 3c). Interestingly, cloudy densities were detected in most class averages, at descrete positions along the filaments. These structures resemble the appearance of the Ig-domains in paracrystalline fibers [41]. To analyze the repeating pattern, we reconstituted the filaments *in silico*, as explained above. The reconstituted filaments exhibit an alternating Ig-fold spacing of 16 ± 2 nm and 28 ± 2 nm, while the wild-type *Ce*-lamin filaments show an alternating Ig-fold spacing of 28 ± 2 nm and 21 ± 2 nm (Figure 3d) [19].

**Figure 4.**
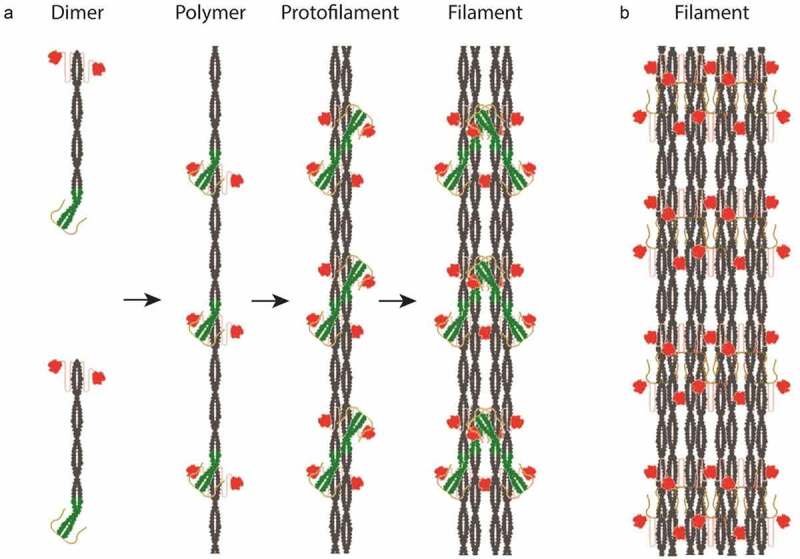
The assembly of *C. elegans* lamin filaments in the presence and absence of helix 1A. (a). Lamin dimers assemble into head-to-tail polymers, which laterally assemble in an antiparallel fashion into protofilaments. *In vitro* assembled *Ce*-lamin is further assembled into ~8-10 nm thick filaments. Helix 1A in green, helices 1B and 2 in gray, flexible tail in pink and Ig-like domain in red. (b). In the absence of helix 1A the assembly continues to form ~20 nm thick filaments in which slight change in the repeat was observed.

The length of helix 1A is 35 aa, which translates into 7 coiled coil heptad repeats, theoretically spanning over ~8 nm. However, deleting helix 1A shortens the repeat of the filament pattern by ~5 nm. This suggests that part of helix 1A contributes to the scaffold of the lamin rod assembly, while its N-terminal stretch may be involved in lateral assembly, presumably inhibiting further interactions and restricting the diameter of the assembled filaments. Based on the presented results, we suggest the following *in vitro* assembly model of *Ce*-lamin filaments (Figure 4). Lamin dimers assemble into head-to-tail polar filaments in which a part of helix segment 1A projects from the axis of the scaffold lamin rod-like structure (Figure 4a). Dimeric lateral assembly forms nonpolar protofilaments, tetrameric in cross-section, similar to the mammalian lamin filaments. *In vitro*, these filaments further assemble into the mature *Ce*-lamin filament. Upon removal of helix 1A (Figure 4b), additional lateral interactions between protofilaments result in thicker final filaments, suggesting that helix 1A is also involved in the assembly control of *Ce*-lamin filaments.

*In vitro* analysis of lamin filaments is of major importance to understand lamin biology and the contribution of individual segments to the overall mature filament assembly. Determining the high-resolution structure of the assembled filaments would significantly improve the understanding of the effect of point mutations on lamin assembly and filament structure. High-resolution structural analysis will likely need to be conducted using *in vitro* experimental approaches, due to the complexity of the components and the crowdedness of the nuclear envelope. In this work, we showed that assembly of *Ce*-lamin is highly dependent on helixes 2 and 1B. However, filaments may form in the absence of the other protein domains. Our results suggest that in *Ce*-lamin, helix 1A contributes partially to the filament scaffold and is also involved in the regulation of the mature filament assembly. Interestingly, the head and tail domains do not play a role in the filament assembly, but may contribute to the interactions with other lamina components and regulators.

## Supplementary Material

Supplemental Material
