# Supplementary material for: Filament assembly of the C. elegans lamin in the absence of helix 1A
Source: Nucleus. 2022 Feb 7;13(1):49–57. doi: 10.1080/19491034.2022.2032917 (PMC8824219; doi:10.1080/19491034.2022.2032917)
Supplement: Supplemental Material [file KNCL_A_2032917_SM1095.pdf]

**Supplementary Information**

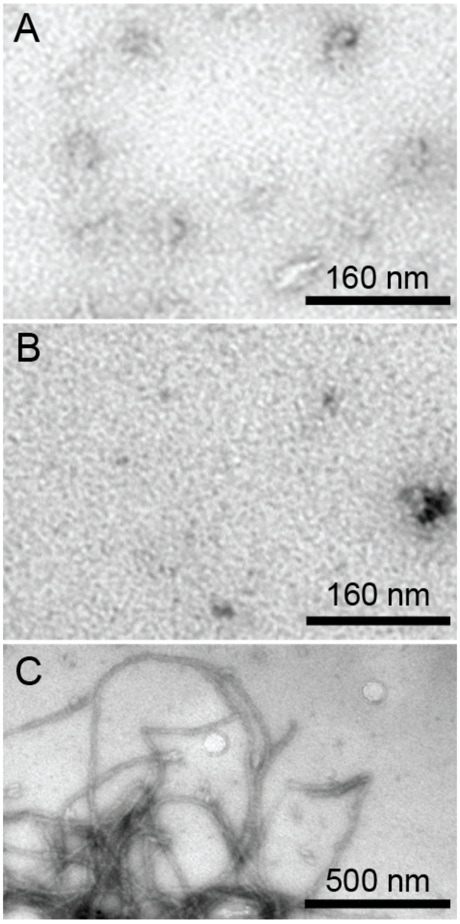


**Figure S1. Negatively stained EM images of *Ce*-lamin lacking helices 1B, 2 and the ΔHead-ΔC15 mutant.** No filaments were detected by negative stained electron microscopy analysis of Ce lamin Δcoil 1B and Δcoil 2 (A and B respectively). C. *Ce*-lamin that lack the head and the last 15aa (ΔHead-ΔC15) assemble into filaments.

## Table 1

Mutagenesis PCR was carried out using the QuikChange Lightning Site-Directed Mutagenesis Kit (Agilent Technologies). Primers were ordered from Microsynth. When applicable, the AgeI restriction site is listed in bold. The AgeI restriction site was introduced and eventually removed.

| | | |
|---|---|---|
| TGAATTCAGCTAATCATCGGATC | ACGGTGATAGAAGAAACAG | Insert STOP codon after Ig-fold |
| ATTTGGCTCAACGCTTCTAGAAACT**ACCGGT**TCACGTCTTCAAG | CTTGAAGACGTGA**ACCGGT**AGTTTCTAGAAGCGTTGAGCCAAAT | Insert AgeI site between head and coil 1A |
| GGTTCAAATTCGCGACATCGAAGT**ACCGGT**TGTTGAAAAGAAAGAGAAG | CTTCTCTTTCTTTTCAACA**ACCGGT**ACTTCGATGTCGCGAATTTGAACC | Insert AgeI site between coil 1A and linker |
| CATTTGGCTCAACGCTTCTAGAAACTTGTTGAAAAGAAAGAGAA | TTCTCTTTCTTTTCAACAAGTTTCTAGAAGCGTTGAGCCAAATG | Remove AgeI site and extra base pairs in Ce-lamin Δcoil 1A |
| GCTCAACGCTTCTAGAAACTGTTGAAAAGAAAGAGAAGTC | GACTTCTCTTTCTTTTCAACAGTTTCTAGAAGCGTTGAGC | Remove extra base pairs in Ce-lamin Δcoil 1A |
| TTGAAAAGAAAGAGAAGTCAAACACCGGTTTGGCCGATCGCTTC | GAAGCGATCGGCCAAACCGTTGTTTGACTTCTCTTTCTTTTCAA | Insert AgeI site between linker and coil 1B |
| CAACAGCACAAGGGAGAA**ACCGGT**CTTGAAGAAGTTCGTCAC | GTGACGAACTTCTTCAAG**ACCGGT**TTCTCCCTTGTGCTGTTG | Insert AgeI site between coil 1B and linker |
| GAAAAGAAAGAGAAGTCAAACCTTGAAGAAGTTCGTCACAAG | CTTGTGACGAACTTCTTCAAGGTTTGACTTCTCTTTCTTTTC | Remove AgeI site in *Ce*-lamin Δcoil 1B |
| TTTGGCTCAACGCTTCTAGAAACTTCTTGAAGAAGTTCG | CGAACTTCTTCAAGAAGTTTCTAGAAGCGTTGAGCCAAA | Remove AgeI site and extra base pairs in Ce-lamin Δcoil 1 |
| CTCAACGCTTCTAGAAACTCTTGAAGAAGTTCGTCACA | TGTGACGAACTTCTTCAAGAGTTTCTAGAAGCGTTGAG | Remove extra base pairs in Ce-lamin Δcoil 1 |
| GAGTATCAATCTAAGCTTCAA**ACCGGT**GATCAAATCGAAGAGATGCGT | ACGCATCTCTTCGATTTGATC**ACCGGT**TTGAAGCTTAGATTGATACTC | Insert AgeI site between linker and coil 2 |
| TGAGGGTGAGGAGGAG**ACCGGT**CGTCTCAATCTTACTC | GAGTAAGATTGAGACG**ACCGGT**CTCCTCCTCACCCTCA | Insert AgeI site between coil 2 and tail |
| GAGTATCAATCTAAGCTTCAACGTCTCAATCTTACTCAGG | CCTGAGTAAGATTGAGACGTTGAAGCTTAGATTGATACTC | Remove AgeI site in *Ce*-lamin Δcoil 2 |
| GAGAGACATATGTTGACTTCACTCAACAG | GAGAGACTCGAGTGTACACATGATGGAACAACGATCGGC | Primers to generate *Ce*-lamin headless |
| GAGAGACATATGTCATCTCGTAAAGGTACTCGTAGTTCTCG | GAGAGATGTACAGAGACGCTCCTCCTCACC | Primers to generate *Ce*-lamin Δtail |
